# Volatiles and Antioxidant Activity of Citrus Fiber/Blackberry Gels: Influence of Sucrose and Trehalose

**DOI:** 10.3390/plants10081640

**Published:** 2021-08-10

**Authors:** Mirela Kopjar, Ivana Ivić, Ivana Buljeta, Ina Ćorković, Josipa Vukoja, Josip Šimunović, Anita Pichler

**Affiliations:** 1 Faculty of Food Technology, Josip Juraj Strossmayer University, F. Kuhača 18, 31000 Osijek, Croatia; iivic@ptfos.hr (I.I.); ivana.buljeta@ptfos.hr (I.B.); ina.corkovic@ptfos.hr (I.Ć.); jjosipa.vukoja@gmail.com (J.V.); anita.pichler@ptfos.hr (A.P.); 2 Department of Food, Bioprocessing and Nutrition Sciences, North Carolina State University, Raleigh, NC 27695-7624, USA; simun@ncsu.edu

**Keywords:** citrus fiber, blackberry juice, sucrose, trehalose, volatiles, phenolics

## Abstract

Citrus fiber/blackberry gels (CBg) can be used for the preparation of various bakery products as well as confectioneries. The objective of this study was to evaluate the influence of the type of disaccharides (sucrose or trehalose) and their percentages (10% or 20%) on volatile compounds as well as phenolics, antioxidant activity and color of formulated CBg. Additionally, CBg were stored at room temperature for 3 months to evaluate their stability. Both disaccharides type and their percentage affected the investigated parameters. Sucrose had a higher positive impact on volatiles after formulation and storage of CBg, while trehalose had a higher positive impact on total phenolics. Amounts of phenolics increased with the increase of disaccharides amount, while the behavior of volatiles also depended on volatiles’ properties. Results of this study emphasized the importance of the adequate choice of ingredients for the formulation of high-quality fruit products.

## 1. Introduction

A plant-based approach for the formulation of semi-prepared products is on the rise. Citrus fiber/blackberry gels could be one of the materials which can be further used for preparation of different bakery products as well as confectioneries. Citrus fruits can be used for the production of different types of products, such as juices, canned fruits or preserved fruits. After production, large amounts of by-products are left over which can be used for the production of dietary fibers, essential oils, pigments or bioactive compounds [1,2]. Production of functional fibers, which can be further used as thickeners, oil carriers, texturizers and moisture retention agents in different food products such as yogurts, ice creams, sauces, dressings, beverages, meat and bakery products, can be quite beneficial [2,3,4,5,6,7]. These benefits can be attributed to the properties of citrus fibers, such as high internal surface area, apparent viscosity, water-holding capacity and binding water to reduce syneresis during food processing [8,9]. In addition to their technological properties, dietary fibers are known for their various health benefits, such as decreasing the risk of development of coronary heart disease, hypertension, diabetes, obesity and some gastrointestinal disorders [10]. In this research, the possible application of citrus fiber for the formulation of gels as semi-prepared products was investigated. Flavor and color are very important quality properties of any food product, as well as of semi-prepared products. When these products are incorporated into the final product, they affect its overall quality. Blackberry juice and disaccharides (sucrose and trehalose) were chosen for the preparation of gels in combination with citrus fibers. Blackberries were chosen as the source of volatiles and phenolics. These fruits are recognized for their specific and pleasant flavor [11,12]. In addition, they provide a rich source of phenolics, which are responsible for their organoleptic attributes and nutritional value, and are also known for their health benefits due to antioxidant properties [13,14]. Two disaccharides have been chosen for this study, sucrose and trehalose. Sucrose is established as a commonly used saccharide in fruit product formulations, while the utilization of trehalose in the formulation of fruit products has been increasing. Sucrose and trehalose’s impact on volatiles and phenolics in different fruit products has been investigated in numerous studies [15,16,17,18,19,20,21,22,23,24,25,26,27,28,29,30,31]. It was concluded that disaccharides’ effect on fruit product quality depended on its type as well as the amount. The objective of this study was the formulation of gels based on citrus fiber, blackberry juice and disaccharides. Sucrose and trehalose were used in order to investigate their impact on volatiles, phenolics, antioxidant activity and color of formulated gels. To evaluate the impact of their amounts on the investigated parameters, disaccharides were added in the amounts of 10% and 20%. Subsequently, formulated gels were stored for 3 months to investigate their storage stability.

## 2. Results and Discussion

Citrus fiber/blackberry gels were prepared from citrus fiber and blackberry juice with the addition of disaccharide, sucrose or trehalose, in amounts of 10% or 20%, in order to investigate the impact of disaccharides types and amounts on the contents of volatiles, total phenolics and proanthocyanidins, as well as antioxidant activities and color parameters.

### 2.1. Volatiles of Citrus Fiber/Blackberry Gels

The flavor profile of the product depends on the flavors of each ingredient that was used for preparation. In our case, sources of volatiles were blackberry juice and citrus fiber. Additionally, some volatiles can be formed during processing. Identified individual volatiles in blackberry juice, citrus fiber and citrus fiber/blackberry gels, molecular weights, hydrophobicity, vapor pressure and odor descriptions are presented in Table 1. In blackberry juice, 19 volatiles were identified, while for citrus fiber, there were 28 and in gels, 29 volatiles. Some of those volatiles were identified only in citrus fiber, such as furaldehyde, 1-octen-3-ol, benzaldehyde, methyl heptenone, m-cymene, 3-octen-2-one, 3,5-octadiene-2-one, myrtenal, 2-butyl-2-octenal, decanol, trans-carveol and carvone, but some of them were also formed during the preparation of gels, such as benzyl alcohol, 2-heptenal, 1-octen-3-one, 6-methyl-5-hepten-2-one and phellandral.

The amounts of volatiles in blackberry juice, citrus fiber and citrus fiber/blackberry gels after preparation with different disaccharides are presented in Table 2. Types of disaccharides and their percentages had an impact on the amounts of volatiles in gels. All volatiles were divided into four groups: alcohols, acids, aldehydes and ketones and terpenes. Five alcohols were identified in gels. Benzyl alcohol was not detected in blackberry juice and in citrus fiber, and thus this volatile was formed during the preparation of gels, probably from benzaldehyde that was identified in citrus fiber but not in juice and gels. Benzyl alcohol was identified in a higher amount when 20% of disaccharides were used for the preparation of gels. Phenethyl alcohol was found in juice, and was retained in gels after their preparation. The highest amount of this alcohol was determined in gels with 20% of sucrose and the lowest with 10% of the same disaccharide. There was no difference between the two gels prepared with trehalose. The remaining alcohols, 2-ethylhexanol, 1-octanol and perillyl alcohol, were identified in all samples. 2-Ethylhexanol and 1-octanol were determined in higher amounts in gels prepared with sucrose regardless of its percentage. The highest amount of perillyl alcohol was determined in gel with 20% of sucrose and the lowest with 10% of the same disaccharide. Trehalose amount did not have an impact on the amount of this alcohol. Two acids were identified in gels. Hexanoic acid was determined on citrus fiber and in gels. Double the amount of this acid was determined in gels in comparison to citrus fiber. By comparing the influence of percentages of disaccharides, it can be noted that the higher amount of this acid was determined when 10% of disaccharides were used for gel preparation. On the other hand, nonanoic acid was identified in blackberry juice in a much higher amount than in prepared gels. Application of sucrose during the preparation of gels caused higher retention of this acid. Aldehydes and ketones were the most abundant group of volatiles identified in all samples. The most volatiles from citrus fiber lost during the preparation of gels belonged to this group of volatiles (furaldehyde, benzaldehyde, methyl heptenone, 3-octen-2-one, 3,5-octadiene-2-one and 2-butyl-2-octenal). In addition, 2-heptenal, 1-octen-3-one and 6-methyl-5-hepten-2-one formed through preparation. 2-heptenal was formed in higher amounts in gels prepared with sucrose. Regardless of disaccharides type, a higher amount of this volatile was formed when 20% of disaccharide was used for preparation. A similar tendency as a result of gel formation was observed for 1-octen-3-one. Type of disaccharide did not have an effect on the formation of 6-methyl-5-hepten-2-one, only their amount, with this volatile formed in higher amounts in gels with 20% of disaccharide. Hexanal, octanal, 2-nonenal and decanal were identified in citrus fiber, and their amounts decreased during the preparation of gels. The highest retention of hexanal was achieved with the lower amounts of disaccharides, and the type of disaccharide did not affect the retention of this volatile. Octanal was identified in higher amounts in gels with sucrose. These gels also had higher amounts of 2-nonenal, but the percentage of disaccharides did not have an impact on the retention of this aldehyde during processing. On the other hand, decanal was determined in higher amounts in gels with trehalose. Lily aldehyde and 2,4-nonadienal were identified in blackberry juice, and their amount significantly decreased during the preparation of gels, but both of them were better retained in gels with sucrose. Heptanal, 2-octenal, 2-decenal and geranyl acetone were volatiles identified in blackberry juice and in citrus fiber as well as in gels. During the preparation of gels, all of these volatiles were retained in higher amounts in sucrose-containing gels. Carvone, m-cymene and trans-carveol were identified only in citrus fiber, and those terpenes were lost during the preparation of gels. Guaiacol, trans-verbenol, nerol and γ-ionone were identified in juice and gels but in lower amounts. Trans-verbenol and γ-ionone were retained better in gels prepared with sucrose, while nerol was better retained in gels with trehalose. In blackberry juice, citrus fiber and gels, D-limonene, linalool, α-ionone and β-ionone were determined. D-limonene was the most abundant volatile in citrus fiber and also in gels. Higher retention of D-limonene was achieved when sucrose was used for the preparation of gels. Those gels also had higher amounts of α-ionone and β-ionone, while linalool was determined in higher amounts in gels with trehalose.

The amounts of volatiles have also been determined after storage (Table 3). All alcohols and acids were determined in higher amounts in gels with sucrose. Aldehydes and ketones were the most stable volatiles during storage, but only hexanal was determined in higher amounts in gels with trehalose. Terpenes, linalool, nerol and α-ionone were determined in higher amounts in gels with trehalose. The most abundant volatile after storage as well as after gel preparation was D-limonene, which was evaluated in the highest amount in sucrose-containing gels.

In most previously published studies [15,16,17,18,19,21,28,29,30] conducted on the effect of trehalose on volatile compounds in different fruit matrices (strawberry cream fillings, orange jelly, strawberry puree, tart cherry puree, pear puree, tart cherry juice, pear cubes), a positive effect of trehalose on the overall retention of volatiles was observed, but in this study, we observed a different trend. In previous studies, it was noted that the positive effect of trehalose on volatiles depended on the properties of volatile compounds, and the matrix composition as well as on the conditions during processing and storage, which resulted in different interactions between components. Generally speaking, in this study, the positive effect of sucrose on volatile compounds was more pronounced than for trehalose. A study on the effect of 5% and 10% addition of sucrose or trehalose on the profile of tart cherry puree volatiles similarly revealed that both disaccharides type and the percentages added had an impact on tart cherry volatile compounds [29], but trehalose had a more positive effect than sucrose. An investigation of sucrose and trehalose effects in amounts of 5%, 10% and 20% on benzaldehyde, benzyl alcohol and 2-hexenal in freeze-dried tart cherry puree showed that the concentrations of those volatiles depended on disaccharide types and their amounts. Additionally, a study on more complex matrices such as strawberry cream fillings (freeze-dried and evaporated) revealed that the retention of strawberry volatiles depended on disaccharides type (sucrose or trehalose) and their amount (3%, 5% and 10%) as well as on the process conditions [18,21]. In the previous studies dealing with the dependence of volatiles’ amount on disaccharide percentages, no increase or decrease of volatiles’ amount was detected as a rule [18,21,29]. Similar results were observed in our study, illustrating that the interactions between components play a very important role in the behavior of volatiles in the fruit matrix. Additionally, competition between components for binding sites needs to be accounted for, since it has been proven that this competition could be a significant factor in the retention of volatiles, especially when they are added as mixtures of compounds [18,21,32,33].

Flavor profiles of products do not depend only on the amounts of volatile compounds but also on their odor threshold, therefore odor activity values (OAVs) were calculated to further elucidate the contribution of individual volatiles to the overall flavor. OAVs were calculated as ratios of volatile concentrations to thresholds [34]. A summary of OAVs of blackberry juice, citrus fiber and gels (after preparation and after storage) based on published odor thresholds is provided in Table 4 and Table 5. In blackberry juice, there were 8 volatiles with OAV higher than 1. The extremely high OAVs (over 10) were observed for γ-ionone, β-ionone and α-ionone (59.71, 25.80 and 21.83, respectively), which are described as berry flavor compounds and 2-decenal (28.5). Fourteen volatiles had OAVs over one in citrus fiber, with eight of them with extremely high OAVs. Especially high OAVs were found for 2-nonenal, D-limonene, 1-octen-3-ol, octanal and β-ionone (143.5, 60.32, 50.28, 5.56 and 34.5, respectively). Comparing OAVs of gels, it was observed that 12 volatiles had values over 1, from which 7 volatiles had extremely high OAVs. 1-octen-3-one had the highest OAV (over 6000). From the aldehyde group of volatiles, 2-nonenal (approximately 69 for trehalose gels and 80 for sucrose ones), octanal, 2-decenal (approximately 11 for trehalose gels and 19 for sucrose ones) and 2-octenal (approximately 10 for trehalose gels and 13 for sucrose ones) were detected with high OAVs. D-limonene was the terpene with the highest OAV in gels (approximately 36.5 for trehalose gels and 50 for sucrose ones), followed by γ-ionone (approximately 36.5 for trehalose gels and 47 for sucrose ones) and β-ionone (approximately 26 for trehalose fillings and 34 for sucrose ones).

During storage, OAVs changed as expected since the amounts of volatiles changed. 1-octen-3-one, characterized by a fruity flavor note, had an extremely high OAV in all gels, and it was determined that for this volatile, OAVs increased over the storage period. 2-Nonenal and octanal (volatiles with green flavor note) and 2-decenal and 2-octenal (volatiles with fatty flavor note) also had high OAVs. For D-limonene (citrus flavor note), OAV significantly decreased, while OAVs of γ-ionone and β-ionone were relatively similar to values after preparation.

**Figure 1:**
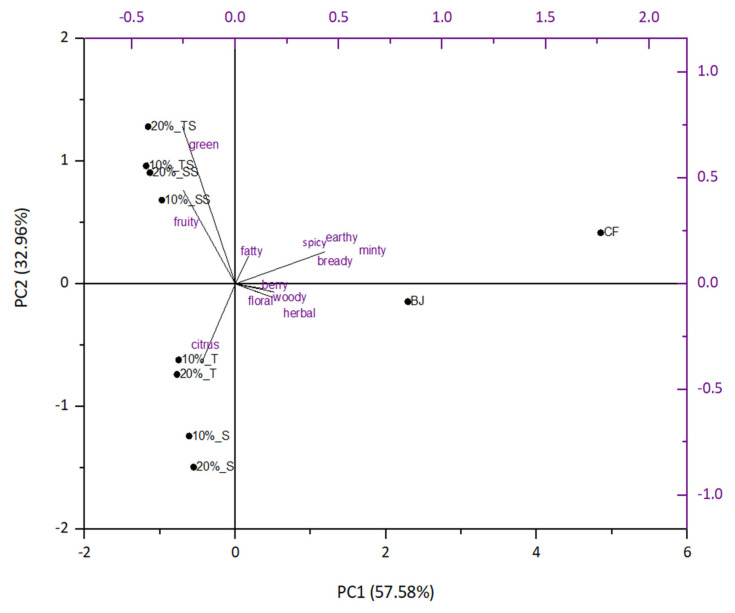
PCA analysis of citrus fiber/blackberry juice gels.

Flavor compounds were divided according to their specific flavor note descriptors, and their percentage contributions to the overall flavor profile of blackberry juice, citrus fiber and gels were calculated. In this work, 12 series were established: citrus, floral, fruity, green, fatty, berry, woody, herbal, earthy, minty, bready and spicy. Results were used for PCA analysis (Figure 1). PC1 accounted for 57.58% of total variance and PC2 for 32.96%. Citrus fiber/blackberry gels were on the left side of the PC1, but they were separated. All gels were on the negative side of PC2 after preparation and on the positive side after storage. Gels after preparation were more characterized by their citrus flavor note. After storage, citrus flavor note decreased while green and fruity flavor notes were more pronounced.

### 2.2. Phenolics, Antioxidant Activity and Color of Citrus Fiber/Blackberry Gels

Total phenolics content and proanthocyanidins content of citrus fiber/blackberry gels are presented in Table 6. From the results, it can be seen that both parameters, type of disaccharides and their amounts, had an impact on total phenolics as well as proanthocyanidins. After preparation of gels, the highest amount of total phenolics was found in the gel prepared with 20% of trehalose (5.233 g/100 g) and the lowest in gel prepared with 10% of sucrose (4.652 g/100 g). Gels prepared with 20% of sucrose and 10% of trehalose had an equal amount of phenolics (4.94 g/100 g). Throughout the storage period, a decrease of total phenolics occurred. Gels prepared with 20% of trehalose (4.558 g/100 g) and 10% of sucrose (4.133 g/100 g) retained the trend that was obtained during preparation, i.e., these samples also had the highest and lowest total phenol contents, respectively. For the other two samples, a different trend was observed. The gel with 20% of sucrose had a slightly lower content of total phenolics (4.504 g/100 g) than the gel with 20% of trehalose, while the gel with 10% trehalose had a lower content than the previously mentioned samples (4.354 g/100 g). Results of the evaluation of proanthocyanidin contents revealed that both gels with trehalose had a higher amount of those compounds (156.85 and 140.46 mg/100 g) than gels with sucrose (129.78 and 120.33 mg/100 g). Additionally, it was evident that samples prepared with the higher disaccharides amount had the higher amount of proanthocyanidins. The highest proanthocyanidins content, after storage, remained in gels with trehalose, but there was no difference between samples prepared with 10% or 20% of this disaccharide (121 mg/100 g). However, in the case of sucrose, samples prepared with a higher amount had higher proanthocyanidins content. Our results are in agreement with other studies that showed similar results on the protection of phenolics, flavonoids and anthocyanins by disaccharides’ addition under different conditions during processing and storage, and emphasized that trehalose had a higher protective effect [18,20,21,22,23,24,25,26,27,30]. As was the case in our study, Kopjar et al. [18] also showed that with the increase of trehalose addition (3%, 5% and 10%) to strawberry cream fillings (evaporated and freeze-dried), an increase of phenolics and anthocyanins occurred, and this trend was retained during storage. In the study dealing with freeze-dried tart cherry puree and investigation of the influence of sucrose and trehalose in amounts of 5%, 10% and 20%, results showed that with the increase of disaccharides amounts, a decrease of total phenolics, flavonoids and anthocyanins occurred, but the positive effects on those phenolics were more pronounced when trehalose was used as an additive [23].

Antioxidant activities of citrus fiber/blackberry gels prepared with different disaccharides after preparation and storage are presented in Table 6. Antioxidant activities of gels were evaluated using DPPH, ABTS, FRAP and CUPRAC methods, and different results were obtained. Antioxidant activity evaluated by the DPPH method revealed that gels with higher amounts of disaccharides had higher antioxidant activities than gels with lower amounts. Comparing values, gels with 20% of sucrose had the highest antioxidant activity and gels with 10% had the lowest. During storage, antioxidant activity decreased and gels with trehalose had higher antioxidant activity than gels with sucrose. In addition, there was no difference in antioxidant activity between gels prepared with different amounts of disaccharides. Using the ABTS method, a different trend in antioxidant activity was observed. Gels with trehalose had higher antioxidant activities than gels with sucrose, and the impact of disaccharides’ amount was not observed. In contrast to all other used methods, an increase of antioxidant activity occurred after storage. As was observed after preparation, gels with trehalose had higher antioxidant activity than sucrose gels. Antioxidant activity evaluated with the FRAP method also revealed that after preparation, gels with trehalose had higher antioxidant activity than those prepared with sucrose. Amount of added trehalose had no effect on antioxidant activity, while gels with 20% of sucrose had a higher value than the ones with 10% of sucrose. After storage, values of antioxidant activity decreased, and the trend observed after preparation remained equal. CUPRAC antioxidant activity revealed that there was no difference between gels with trehalose, and these samples had higher values of antioxidant activity than those prepared with sucrose. After storage, antioxidant activity decreased, but the same trend as after preparation was retained, as was the case with the FRAP method. To explain the occurrence of the higher post-storage antioxidant activity results obtained by the ABTS assay, the nature of ABTS and DPPH radicals were compared. DPPH reacts more slowly with electron and hydrogen donors, which can lead to underestimation of fast reactors. ABTS also reacts with electron and hydrogen donors, but DPPH is much more selective in its reaction with hydrogen donors. ABTS can overestimate the antioxidant activity because it reacts with any hydroxylated aromatics regardless of the real antioxidant potential. Flavonoids that do not have OH groups on the B-ring and aromatic acids that have only one OH group in their structure will not react with DPPH, whereas this is not the case with ABTS [35,36]. Food is a complex matrix in which various interactions between individual ingredients occur that can lead to increased or decreased antioxidant activity. In general, oxidation of phenolics leads to a reduced antioxidant activity, which can be assumed to have occurred with gels during storage (if the ABTS assay was omitted). Furthermore, some studies suggested that partially oxidized phenolics may show higher antioxidant activity than non-oxidized ones [37,38,39,40,41]. It can be concluded that the application of different methods for determining antioxidant activity led to different results due to the differences in the selectivity of methods and mechanisms of their action.

Color parameters of citrus fiber/blackberry gels after preparation and after storage are presented in Table 7. Taking into account the gels after preparation, the highest value of L* belonged to gels with 10% of sucrose (35.79), and the lowest to gels with 20% of trehalose (32.32). After storage, the L* value increased for all samples, and the highest value was again found in gels with 10% of sucrose (39.02), while the lowest value was found in gels with 20% of sucrose (35.96). The highest a* value in the samples after preparation had a gel with 10% of trehalose (13.50), and the lowest gel with 20% of sucrose (12.14). The value of a* after storage was lower in all gels, which indicated a decrease in the intensity of the red color of samples after storage. In the gels after storage, the highest a* value was found for a gel with 20% trehalose (7.76), and the lowest value for a gel with 20% sucrose (6.72). Low positive values of the b* parameter indicated the presence of a small amount of yellow in the samples after preparation. The results showed that gels with 20% sucrose had the lowest b* value (3.92), and gels with 10% trehalose had the highest (4.33). After storage, there was an increase in b* value in all samples, and the highest b* value was found for a gel with 10% sucrose. The lowest change in b* value was observed for a gel with 20% sucrose. Color changes (ΔE) of samples after storage versus samples after the preparation were calculated using the L*, a* and b* values. The largest color change was observed for a gel with 10% sucrose (7.39). The value of °h of samples after preparation ranged from 17.29 to 18.46. It was observed that the °h value increased drastically after storage. Gels with 10% sucrose had the highest °h value both before (18.46) and after storage (45.93). Before storage, gels with 10% trehalose had the highest C* value (14.18), and gels with 20% trehalose had the lowest (12.76). During storage, there was a decrease in C* value in all samples. Highest and lowest C* values after storage were observed in the same samples as after preparation.

Retention or loss of volatile and phenolic compounds depended on the composition of gels and interactions between those compounds. The main difference between gels was in disaccharides and their amounts. Disaccharides used in this study were two chemical isomers, sucrose and trehalose. As proven in other studies, as well as in this one, their behavior in a complex matrix can be different. The behavior of those disaccharides caused by their interactions affected the investigated parameters differently. It is well-known that water has a very specific and important role as a solvent and a reactant in many chemical reactions in foods. Those reactions together with oxidation and molecular mobility can cause degradation of sensitive compounds [42]. Sucrose and trehalose showed different behaviors in model water solutions, which are much simpler systems than fruit matrices. Trehalose has a higher effect on water structure since it has a higher affinity for water and can bind with a higher number of water molecules. Additionally, trehalose forms larger clusters in water than sucrose. Based on hydration number, radius of gyration, glycosidic dihedral angles and cluster formation, it was determined that trehalose–water solutions were more homogeneous than sucrose–water solutions [43]. Trehalose’s impact on water molecules was also proven by investigation of single-particle and collective rotation and translation of water, in which it was concluded that trehalose had a slightly greater effect on these properties than sucrose [44]. In comparison to sucrose, it was observed that trehalose had a stronger perturbing effect on the water structure and that water molecules interacted slightly differently with the different atomic sites of these two disaccharides [45]. A study on water dynamics revealed that trehalose induced slightly stronger retardation of this parameter than sucrose [44], and combined with the development of steric hindrance, it can slow down the nucleophilic attack of water on sensitive compounds [46]. Trehalose and unsaturated compounds can form stable intramolecular complexes which can also contribute to the positive effect of trehalose on phenolics and some volatiles [47,48,49].

## 3. Materials and Methods

### 3.1. Chemicals

Trolox, 2,4,6,-tri (2-pyridyl)-s-triazine (TPTZ), gallic acid, procyanidin B2 and myrtenol were purchased from Sigma-Aldrich (St. Louis, MO, USA). Hydrochloric acid, acetic acid, methanol, sodium carbonate, iron chloride, ammonium acetate and Folin-Ciocalteu reagent were purchased from Kemika (Zagreb, Croatia). 2,2′-azinobis (3-ethylbenzothiozolinesulfonic acid) (ABTS) and 2,2-diphenyl-1-picrylhydrazyl (DPPH) were purchased from Fluka (Buchs, Switzerland). Neocuproine and copper chloride were obtained from Gram-mol (Zagreb, Croatia). Sucrose was purchased from Gram-mol (Zagreb, Croatia), while trehalose was obtained from Hayashibara doo (Okayama, Japan). Citrus fibers were obtained from Biesterfeld AG (Zagreb, Croatia).

### 3.2. Preparation of Citrus Fiber/Blackberry Gels

Blackberry juice was prepared by pressing the berries and filtration of the obtained mass through cheesecloth. Citrus fiber powder contained 76% of dietary fibers, of which 40% was pectin. On a heated magnetic stirrer (80 °C), a mixture of blackberry juice and citrus fiber (5%) was mixed for 10 min. After the mixture was well-homogenized, a disaccharide (sucrose or trehalose) was added in an amount of 10% or 20%. The mixture was homogenized for another 5 min. The obtained hot mixture was filled into heated 40 mL jars and sealed. Samples were left for 24 h and one set of citrus fiber/blackberry gels was used for the evaluation of defined parameters after preparation. The other set of samples was stored for 3 months at room temperature to evaluate stability over the storage period.

### 3.3. Analyses of Volatile Compounds

Volatile compounds from citrus fiber/blackberry gels were extracted using solid-phase microextraction (SPME). For the extraction, 5 g of sample and 1 g of NaCl were weighed in a 10 mL glass vial. SPME fiber coated with divinylbenzene/carboxen/polydimethylsiloxane (DVB/CAR/PDMS) sorbent (50/30 µm, StableFlex™, Supelco, Bellefonte, PA, USA) was used for the extraction of volatiles. The sample vials were conditioned in a temperature-controlled heating module at 40 °C for 45 min and agitated at 350 rpm. After extraction, fiber was removed from samples and volatiles were thermally desorbed in the injector port of the GC. Analysis was conducted on a GC 7890B gas chromatograph (Agilent Technologies, Santa Clara, CA, USA) which was equipped with a 5977A mass spectrometer (Agilent Technologies, Santa Clara, CA, USA). Volatiles were desorbed into a GC injector port at 250 °C for 7 min, in splitless mode. The gas chromatograph was fitted with a HP5 capillary column (60 m × 0.25 mm × 0.25 µm) (Agilent Technologies, Santa Clara, CA, USA). Helium was used as the carrier gas at a flow rate of 1 mL/min at 40 °C. Oven temperature was programmed as follows: the initial temperature of 40 °C was held for 2 min, then 6 °C/min up to 230 °C. Volatiles were identified using a mass selective detector. The detector operated in the m/z range between 45 and 450, and ion source and quadrupole temperature were maintained at 230 and 150 °C, respectively. Compounds were confirmed by matching their mass spectra with the NIST (National Institute of Standards and Technology mass spectral database, Gaithersburg, MD, USA) and through retention time (RT) and retention index (RI) (Table 1). Two repetitions were conducted for each sample. Quantification was conducted by myrtenol as an internal standard and results were expressed as µg/kg.

### 3.4. Extraction of Phenols

Prior to the evaluation of total phenolics, proanthocyanidins and antioxidant activity, the extraction of gels was performed. Two grams of gels and 20 mL of acidified methanol (HCl:methanol ratio was 1:99) were mixed and homogenized. The homogenized mixture was left for 24 h, the mixture was filtered, and the resulting extract was used for the above-mentioned analyses.

### 3.5. Determination of Total Phenolics

The total phenolic contents in the samples were determined by the Folin-Ciocalteu method [50]. Briefly, 0.2 mL of extract and 1.8 mL of deionized water were mixed with 10 mL (1:10) of Folin-Ciocalteu reagent and 8 mL of 7.5% solution of sodium carbonate. Obtained mixtures were left for 2 h in the dark, and the absorbance was read at 765 nm by spectrophotometer (Cary 60, UV-Vis, Agilent Technologies, Petaling Jaya, Selangor, Malaysia). The measurements were performed in triplicates and results were expressed as g of gallic acid equivalents per 100 g of gel (g GAE/100 g).

### 3.6. Determination of Total Proanthocyanidins

Total proanthocyanidins (PAC) concentration was determined colorimetrically using the DMAC method [51]: 0.1 mL of the sample was mixed with 0.4 mL of water and 1 mL of 4-dimethyl-amino-cinnamaldehyde solution. The mixture was left for 30 min and absorbance was measured at 640 nm. The measurements were performed in triplicates and results were expressed as mg of procyanidin B2 equivalents per 100 g of gel (mg B2E/100 g).

### 3.7. Determination of Antioxidant Activity

The ABTS assay followed the method of Arnao et al. [52] with some modifications. Briefly, 0.1 mL of extract was mixed with 3 mL of ABTS (7 mM) reagent and left in the dark. After 95 min, absorbance was measured at 734 nm.

The antioxidant activity of samples was also determined by the radical scavenging activity method using 2,2-diphenyl-1-picrylhydrazyl radical (DPPH) [53]: 0.2 mL of extract was mixed with 3 mL of DPPH (4 mM) solution. After 15 min, absorbance was measured at 517 nm.

The cupric reducing antioxidant capacity assay was carried out according to the method of Apak et al. [54]. Copper chloride, neocuproine and ammonium acetate buffer (pH 7) solution were mixed in a ratio of 1:1:1. Then, 0.2 mL of extract was added to the obtained mixture, followed by 0.9 mL of distilled water. After 30 min, absorbance was recorded at 450 nm.

Additionally, the antioxidant capacity of samples was determined according to the Benzie and Strain method [55] with some modifications: 0.2 mL of extract was added to 3 mL of FRAP (10 mM) reagent to form a mixture. The mixture was incubated for 30 min in the dark, and the absorbance was measured at 593 nm.

For each method for blank, the extract was replaced with water and all measurements were performed in triplicate. Antioxidant activity evaluated by the ABTS, DPPH, FRAP and CUPRAC methods was calculated from the calibration curve with Trolox as a standard and expressed as nmol TE/100 g, except for ABTS (µmol TE/100 g).

### 3.8. Color Measurement and Color Change

Color measurement and color change of citrus fiber/blackberry gels were monitored with a chromometer Minolta CR-400 (Minolta; Osaka, Japan). The Lab system was used for the expression of color. Measured color parameters were as follows: L* (lightness; 0 is black and 100 is white), a* (redness (+) and greenness (−)), b* (yellowness (+) and blueness (−)), C* (the color saturation value—chroma) and °h (the hue angle). L*, a* and b* values were used for the calculation of total color change, ΔE. Measurements were conducted in triplicates.

### 3.9. Statistical Analysis

Comparisons of the obtained results were conducted by analysis of variance (ANOVA) and Fisher’s least significant difference (LSD), with the significance defined at *p* < 0.05. Additionally, principal component analysis (PCA) on volatile compounds was conducted. Software program STATISTICA 13.1 (StatSoft Inc., Tulsa, OK, USA) was used for statistical analyses.

## 4. Conclusions

Results obtained from this research showed that disaccharide types and their amounts used for the formulations of citrus fiber/blackberry gels had an important impact on both volatiles and phenolics. Overall, sucrose had a higher positive impact on volatiles, while trehalose had a higher positive impact on phenolics and proanthocyanidins. Both disaccharides can be used for the formulation of gels. On one hand, using trehalose, we can formulate products with lower sweetness but with higher content of phenolic compounds, while on the other hand, using sucrose, we can formulate products rich in flavor compounds. The obtained gels are semi-products that can be used in the preparation of bakery products and different types of confectionery.

This research provides a starting point for the improvement of existing products and the development of new ones as well. Future studies should be directed to the formulations of final products in order to investigate the influence of gels on their overall quality, such as flavor and color.

## Figures and Tables

**Table 1:** Volatile compounds identified in blackberry juice (BJ), citrus fiber (F) and citrus fiber/blackberry gels (CBg), and their properties.

Volatiles	BJ	F	CBg	RT	RI	CAS No.	MW	LogP (o/w)	VP (mm/Hg)	Odor
hexanal	−	+	+	5.13	800	66-25-1	100.2	1.80	10.888	green
furaldehyde	−	+	−	6.40	827	98-01-1	96.09	0.410	2.234	bready
heptanal	+	+	+	10.76	897	111-71-7	114.2	2.30	3.854	green
benzaldehyde	−	+	−	14.65	956	100-52-7	106.1	1.48	1.270	fruity
2-heptenal	−	−	+	14.95	956	2463-63-0	112.17	2.30	1.823	green
1-octen-3-one	−	−	+	16.46	982	4312-99-6	126.2	2.40	1.063	fruity
1-octen-3-ol	−	+	−	16.50	980	3391-86-4	128.2	2.52	0.531	earthy
methyl heptenone	−	+	−	16.95	985	110-93-0	126.2	1.95	1.277	citrus
6-methyl-5-hepten-2-one	−	−	+	17.11	987	110-93-0	126.2	1.90	1.277	citrus
octanal	−	+	+	18.08	998	124-13-0	128.2	2.70	2.068	green
hexanoic acid	−	+	+	18.54	1005	142-62-1	116.1	1.90	0.158	fatty
m-cymene	−	+	−	19.02	1014	535-77-3	134.2	4.5	1.720	-
D-limonene	+	+	+	19.41	1018	138-86-3	136.2	4.57	0.198	citrus
2-ethylhexanol	+	+	+	19.96	1030	104-76-7	139.0	3.10	0.207	citrus
benzyl alcohol	−	−	+	20.11	1037	100-51-6	192.3	3.2	0.008	fruity
3-octen-2-one	−	+	−	20.24	1036	1669-44-9	126.2	2.179	0.897	earthy
2-octenal	+	+	+	21.49	1054	2548-87-0	126.2	2.60	0.552	fatty
3,5-octadiene-2-one	−	+	−	22.12	1067	38284-27-4	124.2	1.640	0.437	fatty
1-octanol	+	+	+	22.48	1071	111-87-5	130.2	3.00	0.079	green
guaiacol	+	−	+	23.17	1080	90-05-1	124.1	1.32	0.179	green
linalool	+	+	+	23.96	1096	78-70-6	154.3	2.970	0.016	citrus
phenethyl alcohol	+	−	+	24.6	1103	60-12-8	122.2	1.40	0.087	floral
trans-verbenol	+	−	+	25.72	1129	473-67-6	152.2	1.60	0.033	herbal
2-nonenal	−	+	+	27.11	1155	2463-53-8	140.2	3.10	0.256	green
myrtenal	−	+	−	28.64	1183	18486-69-6	150.2	2.98	0.145	spicy
decanal	−	+	+	29.50	1200	112-31-2	156.3	3.80	0.207	floral
2,4-nonadienal	+	−	+	29.86	1205	6750-03-4	138.2	2.70	0.102	fatty
trans-carveol	−	+	−	29.92	1207	18383-51-2	152.2	2.819	0.012	spicy
nerol	+	−	+	30.63	1222	106-25-2	154.3	3.47	0.013	citrus
carvone	−	+	−	31.06	1232	99-49-0	150.2	3.07	0.160	minty
2-decenal	+	+	+	32.23	1255	3913-71-1	154.3	3.70	0.067	fatty
phellandral	−	−	+	32.61	1264	21391-98-0	152.2	2.70	0.098	floral
decanol	−	+	−	32.66	1264	112-30-1	158.3	4.57	0.00851	fatty
perillyl alcohol	+	+	+	33.93	1290	536-59-4	152.2	2.10	0.006	woody
nonanoic acid	+	−	+	33.49	1281	112-05-0	158.2	3.50	0.009	fatty
2-butyl-2-octenal	−	+	−	37.14	1368	13019-16-4	182.3	4.756	0.011	-
α-ionone	+	+	+	38.95	1420	127-41-3	192.3	3.995	0.014	berry
geranyl acetone	+	+	+	39.598	1448	3796-70-1	194.3	3.834	0.016	floral
γ-ionone	+	−	+	40.18	1473	8013-90-9	192.3	3.2	0.008	berry
β-ionone	+	+	+	40.34	1480	14901-07-6	192.3	3.995	0.017	berry
lily aldehyde	+	−	+	41.15	1519	80-54-6	204.3	4.216	0.005	floral

RT—retention time, RI—retention index, MW—molecular weight, VP—vapor pressure at 25 °C, logP—hydrophobicity.

**Table 2:** The amounts of volatiles of blackberry juice (BJ), citrus fiber (F) and citrus fiber/blackberry gels prepared with different disaccharides after preparation.

Volatiles	BJ	F	10%_T	20%_T	10%_S	20%_S
Alcohols						
2-ethylhexanol	20.74 ± 0.01	5.08 ± 0.59	12.39 ± 0.07 ^d^	13.54 ± 0.02 ^c^	14.33 ± 0.05 ^b^	22.93 ± 0.28 ^a^
benzyl alcohol	-	-	21.42 ± 0.04 ^c^	23.03 ± 0.89 ^b^	17.86 ± 0.01 ^d^	25.71 ± 0.37 ^a^
1-octanol	11.90 ± 0.11	23.84 ± 1.54	16.45 ± 0.08 ^c^	15.58 ± 0.28 ^d^	19.09 ± 0.05 ^b^	20.39 ± 0.09 ^a^
1-octen-3-ol	-	50.28 ± 2.56	-	-	-	-
phenethyl alcohol	47.39 ± 0.48	-	11.77 ± 0.05 ^b^	9.91 ± 0.04 ^c^	11.89 ± 0.11 ^b^	13.99 ± 0.10 ^a^
perillyl alcohol	29.19 ± 1.98	3.44 ± 0.25	3.52 ± 0.08 ^b^	3.59 ± 0.23 ^b^	2.78 ± 0.01 ^c^	4.67 ± 0.10 ^a^
Acids						
hexanoic acid	-	18.12 ± 0.05	36.63 ± 3.11 ^a,b^	31.57 ± 2.18 ^b^	38.08 ± 0.56 ^a^	35.51 ± 1.87 ^a,b^
nonanoic acid	24.68 ± 0.18	-	2.31 ± 0.02 ^c^	2.89 ± 0.07 ^b^	4.73 ± 0.11 ^a^	4.66 ± 0.08 ^a^
Aldehydes and ketones						
hexanal	-	183.33 ± 9.2	74.47 ± 0.70 ^a^	60.68 ± 0.14 ^c^	76.41 ± 2.07 ^a^	61.15 ± 0.00 ^b^
furaldehyde	-	40.45 ± 1.71	-	-	-	-
heptanal	0.72 ± 0.00	24.98 ± 0.06	1.20 ± 0.00^c^	1.19 ± 0.00 ^c^	1.42 ± 0.00 ^b^	6.27 ± 0.20 ^a^
benzaldehyde	-	87.76 ± 3.61	-	-	-	-
2-heptenal	-	-	65.81 ± 0.40 ^d^	68.02 ± 0.34 ^c^	69.53 ± 0.03 ^b^	77.13 ± 0.12 ^a^
1-octen-3-one	-	-	30.83 ± 2.37 ^c^	31.30 ± 1.19 ^c^	34.76 ± 0.50 ^b^	38.01 ± 1.91 ^a^
methyl heptenone	-	49.18 ± 0.13	-	-	-	-
6-methyl-5-hepten-2-one	-	-	16.84 ± 0.11 ^b^	18.64 ± 0.01 ^a^	16.95 ± 0.35 ^b^	18.41 ± 0.14 ^a^
octanal	-	31.89 ± 0.41	15.57 ± 0.04 ^d^	21.47 ± 0.33 ^c^	37.89 ± 0.29 ^a^	24.85 ± 0.22 ^b^
3-octen-2-one	-	51.51 ± 4.60	-	-	-	-
2-octenal	10.35 ± 0.13	42.29 ± 3.24	31.98 ± 0.46 ^c^	30.27 ± 2.66 ^c^	39.55 ± 0.14 ^b^	41.07 ± 0.07 ^a^
3,5-octadiene-2-one	-	50.27 ± 3.35	-	-	-	-
2-nonenal	-	14.35 ± 0.03	6.99 ± 0.23 ^b^	6.85 ± 0.05 ^b^	8.17 ± 0.08 ^a^	8.05 ± 1.00 ^a^
myrtenal	-	39.99 ± 0.52	-	-	-	-
decanal	-	12.99 ± 0.71	10.22 ± 0.05 ^a^	10.52 ± 0.07 ^a^	6.23 ± 0.11 ^c^	9.09 ± 0.03 ^b^
2,4-nonadienal	22.12 ± 0.41	-	3.83 ± 0.04 ^c^	3.58 ± 0.25 ^c^	5.28 ± 0.07 ^b^	7.21 ± 0.08 ^a^
2-decenal	11.38 ± 0.25	7.47 ± 0.43	4.29 ± 0.02 ^d^	4.69 ± 0.09 ^c^	7.43 ± 0.22 ^b^	8.22 ± 0.00 ^a^
2-butyl-2-octenal	-	4.39 ± 0.01	-	-	-	-
geranyl acetone	18.68 ± 0.41	7.35 ± 0.10	4.70 ± 0.06 ^d^	5.48 ± 0.13 ^c^	6.16 ± 0.00 ^b^	6.99 ± 0.14 ^a^
lily aldehyde	11.26 ± 0.04	-	2.66 ± 0.07 ^a^	2.90 ± 0.09 ^b^	3.82 ± 0.09 ^c^	5.39 ± 0.13 ^d^
Terpenes						
m-cymene	-	6.99 ± 0.25	-	-	-	-
D-limonene	7.37 ± 0.13	603.15 ± 3.3	368.44 ± 10.35 ^b^	362.97 ± 4.75 ^b^	496.20 ± 5.42 ^a^	505.88 ± 2.74 ^a^
guaiacol	104.17 ± 1.8	-	14.29 ± 0.02 ^b^	12.82 ± 0.02 ^c^	11.35 ± 0.30 ^d^	15.75 ± 0.17 ^a^
linalool	23.39 ± 0.42	11.83 ± 0.21	8.16 ± 0.09 ^a^	7.75 ± 0.14 ^b^	5.33 ± 0.02 ^c^	5.21 ± 0.05 ^c^
trans-verbenol	43.40 ± 0.46	-	4.16 ± 0.07 ^c^	2.88 ± 0.14 ^d^	4.59 ± 0.11 ^b^	5.67 ± 0.03^a^
trans-carveol	-	20.03 ± 0.21	-	-	-	-
nerol	20.25 ± 0.61	-	4.26 ± 0.02 ^b^	4.70 ± 0.21 ^a^	3.90 ± 0.10^c^	4.50 ± 0.05^a^
carvone	-	17.84 ± 1.01	-	-	-	-
phellandral	-	-	3.67 ± 0.02 ^c^	5.02 ± 0.29 ^a^	4.28 ± 0.00 ^b^	5.57 ± 0.00 ^a^
α-ionone	13.10 ± 0.44	1.51 ± 0.02	1.15 ± 0.02 ^c^	1.40 ± 0.03 ^b^	2.99 ± 0.00 ^a^	1.56 ± 0.01 ^b^
γ-ionone	4.18 ± 0.43	-	2.58 ± 0.07 ^b^	2.48 ± 0.05 ^b^	3.35 ± 0.00 ^a^	3.27 ± 0.10 ^a^
β-ionone	2.58 ± 0.11	3.45 ± 0.32	2.52 ± 0.04 ^c^	2.62 ± 0.05 ^c^	3.36 ± 0.03 ^b^	3.59 ± 0.00 ^a^

Within the row, means followed by different superscripts are significantly different at *p* ≤ 0.05 (ANOVA, Fisher’s LSD). S—citrus fiber/blackberry gels with sucrose; T—citrus fiber/blackberry gels with trehalose.

**Table 3:** Amounts of volatiles of citrus fiber/blackberry gels prepared with different disaccharides after storage.

Volatiles	10%_T	20%_T	10%_S	20%_S
Alcohols				
2-ethylhexanol	12.09 ± 0.07 ^b^	9.02 ± 0.09 ^d^	9.93 ± 0.11 ^c^	13.73 ± 0.16 ^a^
benzyl alcohol	11.94 ± 0.06 ^c^	9.10 ± 0.10 ^d^	12.93 ± 0.11 ^b^	17.07 ± 0.08 ^a^
1-octanol	14.65 ± 0.22 ^c^	13.89 ± 0.10 ^d^	20.48 ± 0.07 ^b^	25.15 ± 0.37 ^a^
phenethyl alcohol	9.31 ± 0.08 ^b^	8.67 ± 0.12 ^c^	8.45 ± 0.06 ^c^	12.38 ± 0.01 ^a^
perillyl alcohol	2.32 ± 0.06 ^c^	2.85 ± 0.06 ^b^	2.58 ± 0.15 ^b,c^	4.57 ± 0.02 ^a^
Acids				
hexanoic acid	28.46 ± 1.88 ^a,b^	26.40 ± 0.12 ^b,c^	29.81 ± 1.84 ^a^	24.79 ± 1.35 ^c^
nonanoic acid	1.72 ± 0.01 ^c^	1.70 ± 0.01 ^c^	2.97 ± 0.01 ^b^	4.25 ± 0.24 ^a^
Aldehydes and ketones				
hexanal	66.23 ± 0.38 ^a^	53.73 ± 1.53 ^b^	49.77 ± 0.92 ^c^	47.92 ± 0.63 ^c^
heptanal	-	-	2.67 ± 0.02 ^b^	2.86 ± 0.09 ^a^
2-heptenal	56.08 ± 0.78 ^c^	52.81 ± 0.59 ^d^	78.63 ± 0.34 ^b^	99.35 ± 1.25 ^a^
1-octen-3-one	40.52 ± 0.10 ^b^	37.29 ± 0.02 ^c^	29.12 ± 0.09 ^d^	61.88 ± 1.53 ^a^
6-methyl-5-hepten-2-one	21.13 ± 0.07 ^c^	22.23 ± 0.58 ^b^	10.80 ± 0.01 ^d^	28.66 ± 1.83 ^a^
octanal	16.01 ± 0.10 ^c^	21.25 ± 0.64 ^b^	28.19 ± 0.21 ^a^	29.06 ± 1.93 ^a^
2-octenal	21.98 ± 0.34 ^c^	16.44 ± 0.12 ^d^	30.67 ± 0.45 ^b^	44.68 ± 1.76 ^a^
2-nonenal	5.56 ± 0.02 ^b^	4.73 ± 0.00 ^c^	8.32 ± 0.23 ^a^	8.36 ± 0.03 ^a^
decanal	5.33 ± 0.01 ^d^	6.18 ± 0.10 ^c^	7.49 ± 0.07 ^b^	9.56 ± 0.12 ^a^
2,4-nonadienal	3.71 ± 0.10 ^c^	5.84 ± 0.22 ^b^	5.44 ± 0.04 ^b^	6.65 ± 0.03 ^a^
2-decenal	3.47 ± 0.10 ^c^	3.39 ± 0.02 ^c^	5.98 ± 0.16 ^b^	7.86 ± 0.14 ^a^
geranyl acetone	2.78 ± 0.04 ^c^	2.66 ± 0.03 ^c^	4.41 ± 0.18 ^b^	4.99 ± 0.08 ^a^
lily aldehyde	2.63 ± 0.16 ^c^	2.53 ± 0.19 ^c^	3.57 ± 0.28 ^b^	4.89 ± 0.12 ^a^
Terpenes				
D-limonene	215.25 ± 4.71 ^c^	174.82 ± 0.34 ^d^	278.32 ± 12.99 ^b^	304.63 ± 5.42 ^a^
guaiacol	9.66 ± 0.14 ^c^	9.00 ± 0.15 ^c^	10.19 ± 0.05 ^b^	12.46 ± 0.05 ^a^
linalool	6.89 ± 0.17 ^a^	6.74 ± 0.10 ^a^	3.34 ± 0.03 ^c^	4.14 ± 0.08 ^b^
trans-verbenol	2.88 ± 0.18 ^a,b^	2.71 ± 0.11 ^b^	3.09 ± 0.14 ^a^	2.76 ± 0.06 ^b^
nerol	3.81 ± 0.07 ^b,c^	4.27 ± 0.06 ^a^	3.66 ± 0.07 ^c^	4.02 ± 0.07 ^b^
phellandral	2.03 ± 0.00 ^d^	3.72 ± 0.06 ^b^	3.18 ± 0.09 ^c^	4.38 ± 0.02 ^a^
α-ionone	1.10 ± 0.01 ^b^	1.74 ± 0.27 ^a^	1.03 ± 0.01 ^b^	0.98 ± 0.01 ^b^
γ-ionone	2.02 ± 0.01 ^c^	2.34 ± 0.03 ^b^	2.40 ± 0.00 ^b^	2.82 ± 0.23 ^a^
β-ionone	2.74 ± 0.18 ^a^	2.39 ± 0.02 ^b^	2.29 ± 0.02 ^b^	2.65 ± 0.06 ^a^

Within the column, means followed by different superscripts are significantly different at *p* ≤ 0.05 (ANOVA, Fisher’s LSD). S—citrus fiber/blackberry gels with sucrose; T—citrus fiber/blackberry gels with trehalose.

**Table 4:** Odor activity values (OAVs) of blackberry juice, citrus fiber and citrus fiber/blackberry gels after preparation.

Volatiles	OT (µg/kg)	OAVs					
		BJ	F	10%_T	20%_T	10%_S	20%_S
Alcohols							
2-ethylhexanol	138	0.15	0.04	0.09	0.10	0.10	0.17
1-octen-3-ol	1	0.00	50.28	0.00	0.00	0.00	0.00
benzyl alcohol	100	0.00	0.00	0.21	0.23	0.18	0.26
1-octanol	130	0.09	0.18	0.13	0.12	0.15	0.16
phenethyl alcohol	1000	0.05	0.00	0.01	0.01	0.01	0.01
perillyl alcohol	1660	0.02	0.00	0.00	0.00	0.00	0.00
Acids							
hexanoic acid	1000	0.00	0.02	0.04	0.03	0.04	0.04
nonanoic acid	9	2.74	0.00	0.26	0.32	0.53	0.52
Aldehydes and ketones							
hexanal	20	0.00	9.17	3.72	3.03	3.82	3.06
furaldehyde	3	0.00	13.48	0.00	0.00	0.00	0.00
heptanal	3	0.24	8.33	0.40	0.40	0.47	2.09
benzaldehyde	5000	0.00	0.02	0.00	0.00	0.00	0.00
2-heptenal	13	0.00	0.00	5.06	5.23	5.35	5.93
1-octen-3-one	0.005	0.00	0.00	6166	6260	6952	7602
methyl heptenone	140	0.00	0.35	0.00	0.00	0.00	0.00
octanal	0.7	0.00	45.56	22.24	30.67	54.13	35.50
2-octenal	3	3.45	14.10	10.66	10.09	13.18	13.69
2-nonenal	0.1	0	143.5	69.9	68.5	81.7	80.5
decanal	2	0.00	6.50	5.11	5.26	3.12	4.55
2-decenal	0.4	28.5	18.7	10.7	11.7	18.6	20.6
geranyl acetone	60	0.31	0.12	0.08	0.09	0.10	0.12
Terpenes							
D-limonene	10	0.74	60.32	36.84	36.30	49.62	50.59
guaiacol	20	5.21	0.00	0.71	0.64	0.57	0.79
linalool	6	3.90	1.97	1.36	1.29	0.89	0.87
nerol	290	0.07	0.00	0.01	0.02	0.01	0.02
carvone	6.7	0.00	2.66	0.00	0.00	0.00	0.00
α-ionone	0.6	21.83	2.52	1.92	2.33	4.98	2.60
γ-ionone	0.07	59.71	0.00	36.86	35.43	47.86	46.71
β-ionone	0.1	25.80	34.50	25.20	26.20	33.60	35.90

**Table 5:** Odor activity values (OAVs) of blackberry juice, citrus fiber and citrus fiber/blackberry gels after storage.

Volatiles		OAVs		
	10%_T	20%_T	10%_S	20%_S
Alcohols				
2-ethylhexanol	0.09	0.07	0.07	0.10
benzyl alcohol	0.12	0.09	0.13	0.17
1-octanol	0.11	0.11	0.16	0.19
phenethyl alcohol	0.01	0.01	0.01	0.01
perillyl alcohol	0.00	0.00	0.00	0.00
Acids				
hexanoic acid	0.03	0.03	0.03	0.02
nonanoic acid	0.19	0.19	0.33	0.47
Aldehydes and ketones				
hexanal	3.31	2.69	2.49	2.40
heptanal	0.00	0.00	0.87	0.95
2-heptenal	4.31	4.06	6.05	7.64
1-octen-3-one	8104	7458	5824	12376
octanal	22.87	30.36	40.27	41.51
2-octenal	7.33	5.48	10.22	14.89
2-nonenal	55.60	47.30	83.20	83.60
decanal	2.67	3.09	3.75	4.78
2-decenal	8.68	8.48	14.95	19.65
geranyl acetone	0.05	0.04	0.07	0.08
Terpenes				
D-limonene	21.53	17.48	27.83	30.46
guaiacol	0.48	0.45	0.51	0.62
linalool	1.15	1.12	0.56	0.69
nerol	0.01	0.01	0.01	0.01
α-ionone	1.83	2.90	1.72	1.63
γ-ionone	28.86	33.43	34.29	40.29
β-ionone	27.40	23.90	22.90	26.50

**Table 6:** Total phenolics content (TPC), proanthocyanidins content (PAC) and antioxidant activities of citrus fiber/blackberry gels prepared with different disaccharides after preparation and storage.

Samples	TPC	PAC	DPPH	ABTS	FRAP	CUPRAC
After preparation						
10%_S	4.652 ± 0.005 ^c^	120.33 ± 0.98 ^d^	197.92 ± 1.23 ^d^	1.054 ± 0.038 ^d^	245.71 ± 2.94 ^c^	148.76 ± 0.98 ^d^
20%_S	4.937 ± 0.056 ^b^	129.78 ± 0.45 ^c^	233.91 ± 1.47 ^a^	1.114 ± 0.067 ^d^	253.70 ± 0.41 ^b^	153.71 ± 1.21 ^a^
10%_T	4.946 ± 0.045 ^b^	140.46 ± 1.11 ^b^	204.13 ± 1.21 ^c^	1.478 ± 0.044 ^b^	292.29 ± 1.10 ^a^	170.16 ± 1.05 ^b^
20%_T	5.233 ± 0.044 ^a^	156.85 ± 1.34 ^a^	216.55 ± 1.57 ^b^	1.526 ± 0.099 ^b^	291.05 ± 0.96 ^a^	170.61 ± 1.11 ^b^
After storage						
10%_S	4.133 ± 0.044 ^f^	88.45 ± 0.20 ^f^	160.03 ± 1.37 ^f^	1.248 ± 0.011 ^c^	206.69 ± 1.65 ^e^	134.24 ± 0.97 ^f^
20%_S	4.504 ± 0.031 ^d^	105.56 ± 0.77 ^e^	164.98 ± 1.41 ^f^	1.214 ± 0.038 ^c^	226.42 ± 3.34 ^d^	142.40 ± 1.99 ^e^
10%_T	4.354 ± 0.062 ^e^	120.26 ± 1.73 ^d^	181.45 ± 1.47 ^e^	1.502 ± 0.018 ^b^	254.42 ± 1.87 ^b^	166.80 ± 1.17 ^c^
20%_T	4.558 ± 0.054 ^d^	121.72 ± 1.56 ^d^	184.19 ± 1.61 ^e^	1.654 ± 0.077 ^a^	256.49 ± 1.52 ^b^	167.42 ± 1.01 ^c^

Within the column, means followed by different superscripts are significantly different at *p* ≤ 0.05 (ANOVA, Fisher’s LSD). S—citrus fiber/blackberry gels with sucrose; T—citrus fiber/blackberry gels with trehalose. TPC expressed in g GAE/100 g; PAC expressed in mg B2E/100 g; DPPH, FRAP and CUPRAC expressed in nmol TE/100 g; ABTS expressed in µmol TE/100 g.

**Table 7:** Color parameters (L*, a* and b*) of citrus fiber/blackberry gels prepared with different disaccharides after preparation and storage.

Samples	L*	a*	b*	ΔE	°h	C*
After preparation						
10%_S	35.79 ± 0.03 ^e^	12.90 ± 0.01 ^b^	4.31 ± 0.02 ^e^		18.46 ± 0.09 ^d^	13.60 ± 0.01 ^b^
20%_S	32.95 ± 0.03 ^g^	12.14 ± 0.03 ^c^	3.92 ± 0.01 ^g^		17.92 ± 0.06 ^e^	12.76 ± 0.02 ^c^
10%_T	34.91 ± 0.03 ^f^	13.50 ± 0.05 ^a^	4.33 ± 0.02 ^e^		17.75 ± 0.12 ^e^	14.18 ± 0.05 ^a^
20%_T	32.32 ± 0.01 ^h^	12.95 ± 0.03 ^b^	4.03 ± 0.02 ^f^		17.29 ± 0.07 ^f^	13.56 ± 0.03 ^b^
After storage						
10%_S	39.02 ± 0.03 ^a^	6.85 ± 0.03 ^e^	7.08 ± 0.03 ^a^	7.39	45.93 ± 0.21 ^a^	9.85 ± 0.04 ^d^
20%_S	35.96 ± 0.03 ^d^	6.72 ± 0.03 ^f^	4.79 ± 0.03 ^d^	6.27	35.50 ± 0.14 ^c^	8.25 ± 0.03 ^f^
10%_T	38.19 ± 0.03 ^b^	7.66 ± 0.03 ^d^	5.70 ± 0.03 ^b^	6.84	36.64 ± 0.12 ^b^	9.55 ± 0.04 ^e^
20%_T	36.25 ± 0.03 ^c^	7.76 ± 0.03 ^d^	5.49 ± 0.03 ^c^	6.67	35.28 ± 0.15 ^c^	9.50 ± 0.03 ^e^

Within the column, means followed by different superscripts are significantly different at *p* ≤ 0.05 (ANOVA, Fisher’s LSD). S—citrus fiber/blackberry gels with sucrose; T—citrus fiber/blackberry gels with trehalose. ΔE—change of color of citrus fiber/blackberry gels after storage in relation to gels after preparation.

## Data Availability

Not applicable.
